# 

**Published:** 1882-10

**Authors:** 


### Pamphlets.

---

A case of ovariotomy. By E. Miller, M.D., Florence, S. C. Reprint from the *North Carolina Medical Journal.*

Ten years experience in the treatment of stricture of the urethra by electrolysis. By Robert Newman, M.D., New York. Reprint from the *Medical Record.*

A successful ovariotomy in 1866. Galvano caustic to pedicle, antiseptic dressing, drainage, silver wire ligature left in abdominal cavity. By Robert Newman, M.D., New York. Reprint from the *New England Medical Monthly.*

Nervous control or equilibration. By James T. Searcy, Tuskaloosa, Ala. Reprint from the *Transactions of the Medical Association of the State of Alabama.*

A reply to Dr. Foster Pratt's paper on the "Legal responsibility of surgeons for ununited fractures," read before the Michigan State Medical Society at its annual meeting in May, 1882. By Donald Maclean, M.D.

Life of John M. Briggs, of Boling Green, Kentucky. By W. K. Boling, M.D. Reprint from the *Nashville Journal of Medicine and Surgery.*

The antiseptic treatment of wounds, after operations and injuries. By W. T. Briggs, M.D. Reprint from the *Nashville Journal of Medicine and Surgery.*

Elephantiasis arabum in the Samoan Islands. By Arthur C. Heffenger, M.D., United States Navy.

The presence of the micrococcus in the blood of malignant measles. Its importance and treatment. By John M. Keating, M.D. Reprint from the *Philadelphia Medical Times.*

Semi-annual mortuary report of the city of Savannah. By J. T. McFarland, M.D., health officer, with the compliments of Dr. J. C. LeHardy.

The disease of the Scythians (*morbus feminarum*), and certain analogous conditions. By Wm. A. Hammond, M.D., New York. Read before the American Neurological Association, and reprinted from the *American Journal of Neurology and Psychaiatry.*

---

"WHAT shall we do with Homœopathy?" is the title of a communication in the *Michigan Medical News.*

Let it alone, we say. It will after awhile become old fashioned, and die a natural death without the aid of physicians.

---

THE American Public Health Association will hold its tenth annual session at Indianapolis, Indiana, commencing Tuesday, October 17th, and continuing four days.

